# Thrombotic Thrombocytopenic Purpura Induced by Immune Checkpoint Inhibitiors: A Case Report and Review of the Literature

**DOI:** 10.7759/cureus.11246

**Published:** 2020-10-29

**Authors:** Zeeshan Ali, Muhammad Usman Zafar, Zachary Wolfe, Faisal Akbar, Bradley Lash

**Affiliations:** 1 Hematology and Oncology, Lehigh Valley Health Network, Allentown, USA; 2 Hospital Medicine, Lehigh Valley Health Network, Allentown, USA; 3 Hematology and Medical Oncology, Lehigh Valley Health Network, Allentown, USA

**Keywords:** thrombotic thrombocytopenic thrombocytopenia, immune check-point inhibitors, immunotherapy-related adverse events, caplacizumab

## Abstract

In recent years, the role of immune checkpoint inhibitors (ICI) in cancer treatment has rapidly expanded. In randomized clinical trials, these agents have demonstrated clinical efficacy in extending survival and increasing response rates. Immune-related adverse effects (irAEs) involving various organs have been frequently narrated. Herein, we present a case report of thrombotic thrombocytopenic purpura (TTP) as a rare side effect of nivolumab, plus ipilimumab, in the treatment of metastatic renal cell carcinoma (RCC). A review of the literature for other case reports of TTP during treatment with ICIs was also performed. Our aim is to elucidate the significance of early recognition of this rare adverse effect in patients being treated with this relatively newer class of medications.

## Introduction

Renal cell carcinoma (RCC) is one of the most common malignancies of the genitourinary tract. In 2018, > 400,000 new cases were diagnosed and RCC accounted for > 175,000 deaths worldwide [1].

A significant proportion of patients presents with advanced disease and despite a considerable recent development in diagnostic and therapeutic modalities, survival in this patient population remains low [2]. In the modern era of targeted therapies and immunotherapy, risk stratification nomograms have been developed to assist with upfront treatment decision making in patients with metastatic RCC; the two most commonly employed are Memorial Sloan-Kettering Cancer Center (MSKCC) Motzer Score and International Metastatic RCC Database Consortium (IMDC) Risk Score (0 = favorable, 1 - 2 = intermediate, 3 = poor) [3-4].

Programmed death-1 (PD-1) and cytotoxic T-lymphocyte antigen-4 (CTLA-4) are protein receptors expressed on the surface of T-cells. Their interaction with corresponding ligands on tumor cells and antigen-presenting cells results in T-cell inhibition. Nivolumab and ipilimumab are monoclonal antibodies that block PD-1 and CTLA-4, respectively, and thereby enhance T-cell activation and anti-tumor immune response. Based on the results of a phase III trial, CheckMate 214 (http://www.clinicaltrials.gov/ct2/show/NCT02231749?term=CheckMate+214&draw=2&rank=1), the combination of immune checkpoint inhibitors (ICI), nivolumab and ipilimumab, was granted approval by the Food and Drug Administration (FDA) for use in patients with treatment-naïve, intermediate, or poor-risk advanced RCC [5]. The most common immune-related adverse events (irAEs) reported in phase III trials of these agents include fatigue, pruritis, diarrhea, rash, nausea, increased lipase level, and hypothyroidism [5-6]. A rare side effect of ICI treatment, which was not originally described in these trials but has later been reported in the literature, is thrombotic thrombocytopenic purpura (TTP) [7-8].

We describe a rare case of TTP in a patient with advanced RCC who received treatment with checkpoint inhibitors. We also review the literature for cases of TTP associated with ICI therapy.

## Case presentation

A 46-year-old man was diagnosed with intermediate-risk Stage IV clear cell renal cell carcinoma of the left kidney with pulmonary and cutaneous metastatic disease. Treatment was planned to begin with ipilimumab, 1 mg/kg, and nivolumab, 3 mg/kg every three weeks for four cycles, followed by nivolumab, 240 mg every two weeks, as frontline therapy. CT scans of the chest, abdomen, and pelvis, obtained after three cycles of combined immunotherapy, showed excellent response with a marked reduction in the size of the primary mass and metastatic lesions. Two weeks after receiving his fourth cycle of ipilimumab and nivolumab, the patient presented with intermittent fever, back pain, and hematuria. His outpatient medications included albuterol inhaler and amlodipine tablets. Physical examination did not reveal any abnormal findings.

Laboratory studies revealed acute kidney injury (creatinine 2.61 mg/dL; baseline 1.2 mg/dL), hyperbilirubinemia (total bilirubin 2.1 mg/dL), anemia (hemoglobin 9.5 g/dL; baseline 14.3 g/dL) with reticulocyte count of 8.4% (corrected reticulocyte count 2.4%), and thrombocytopenia (platelet count 12,000/cubic millimeter; baseline 399,000/cubic millimeter). Urinalysis was consistent with hemoglobinuria (3+ blood on the dipstick, no red blood cells (RBCs) on microscopy). Lactate dehydrogenase (LDH) was 1,379 U/L (reference range: 100 - 250 U/L) and haptoglobin was undetectable. A review of peripheral smear showed evidence of microangiopathic hemolytic anemia with consistent schistocytosis across multiple high-power fields and thrombocytopenia. 

Because of high suspicion for TTP, ADAMTS13 activity testing was sent. Plasma exchange (PLEX) was initiated with a single plasma volume per day, along with prednisone, 80 mg (1 mg/kg) daily, and rituximab, 100 mg q weekly. After six days of PLEX, platelets had recovered to 245,000/cubic millimeter, LDH improved to 320 U/L, and the acute kidney injury resolved (creatinine improved to 1.5 mg/dL). PLEX was stopped on Day 7 as platelets remained > 150,000/cubic millimeter for two consecutive days. ADAMTS13 activity level subsequently returned as < 2% (reference value > 66.8%) with an inhibitor titer of > 186 U/mL (reference value < 12 U/mL). The patient continued on a prednisone taper (dose was reduced to 60 mg daily at the time of discharge), and it was planned to complete a total of four doses of weekly rituximab. 

However, labs drawn five days later revealed a platelet count of 7,000/cubic millimeters. The patient was asymptomatic. PLEX was restarted with the continuation of prednisone (60 mg daily) and weekly rituximab. Caplacizumab was initiated at a dose of 11 mg daily administered subcutaneously after completion of each PLEX session. After five days, PLEX was stopped as platelets remained > 150,000/cubic millimeter for two consecutive days. Caplacizumab was continued for 30 days following the last PLEX. The patient completed four weeks of rituximab. Prednisone was weaned down to 10 mg daily over the ensuing 10 weeks. As of his last follow-up visit (about nine months after the first episode of TTP), he has not had a relapse of TTP. Due to the development of immune-mediated TTP, checkpoint inhibitor therapy was permanently discontinued. For metastatic RCC, his treatment was switched to cabozantinib, 40 mg daily. The most recent body imaging has revealed stable disease.

## Discussion

TTP is a life-threatening disorder that involves the widespread formation of microthrombi, rich in von Willebrand factor (vWF) and platelets, which ultimately causes consumptive thrombocytopenia, schistocyte formation, microangiopathic hemolysis, and impaired tissue perfusion [9]. It is characterized by an immune-mediated deficiency of a disintegrin-like and metalloproteinase with thrombospondin type 1 motif, member 13 (ADAMTS13), an enzyme responsible for cleavage of highly thrombogenic, large vWF multimers. Treatment with plasma exchange and rituximab is usually efficacious in preventing irreversible end-organ damage and death; however, this disorder can recur unpredictably. Caplacizumab is a humanized immunoglobulin fragment that targets the A1 domain of vWF and prevents its interaction with platelets.

To our knowledge, only three other cases of TTP caused by checkpoint inhibitors have been reported in the literature [7-8, 10]. Youssef et al. reported a case of TTP that occurred nine days following the administration of one cycle of nivolumab and ipilimumab [8]. TTP has also been described following the use of single-agent immunotherapy with ipilimumab [7] and pembrolizumab [10]. Follow-up body CT scans in our patient, obtained prior to the administration of the fourth cycle of immunotherapy, had shown excellent response to treatment with a significant reduction in tumor burden. Thrombocytopenia with laboratory evidence of microangiopathic hemolysis was observed approximately two weeks after the fourth dose of nivolumab and ipilimumab. Immune-mediated TTP was confirmed by ADAMTS13 activity of less than 10% and a very high inhibitor titer. Relapse occurred within a week after initial stabilization. With the administration of caplacizumab, we were able to achieve a durable response. Presenting symptoms of TTP commonly include fever, petechial rash, and neurologic manifestations, such as slurred speech, headache, and confusion. Elevated serum levels of creatinine and liver transaminases can be seen. In addition to the more commonly described organ-specific irAEs, such as dermatitis, colitis, hepatitis, thyroiditis, and hypophysitis, it is critical to recognize TTP in patients treated with checkpoint inhibitors as prompt initiation of treatment can be lifesaving.

## Conclusions

The combination of nivolumab and ipilimumab has shown a survival benefit in patients with metastatic malignancies. The side effect profile of these agents should be carefully considered since the treatment of such cancers is palliative and not curative. With early recognition of TTP and rapid institution of recommended treatments, we were able to successfully manage this condition in our patient.
